# Metabolic regulation of hepatic PNPLA3 expression and severity of liver fibrosis in patients with NASH

**DOI:** 10.1111/liv.14402

**Published:** 2020-03-01

**Authors:** Francesca V. Bruschi, Matteo Tardelli, Merima Herac, Thierry Claudel, Michael Trauner

**Affiliations:** ^1^ Hans Popper Laboratory of Molecular Hepatology Division of Gastroenterology and Hepatology Internal Medicine III Medical University of Vienna Vienna Austria; ^2^ Division of Gastroenterology and Hepatology Joan and Sanford I. Weill Cornell Department of Medicine Weill Cornell Medical College New York NY USA; ^3^ Clinical Institute of Pathology Medical University of Vienna Vienna Austria

**Keywords:** genetic polymorphism, lipase, lipid stress, non‐alcoholic fatty liver disease

## Abstract

**Background and Aims:**

The genetic *PNPLA3* polymorphism I148M has been extensively associated with higher risk for development and progression of NAFLD towards NASH.

**Methods:**

PNPLA3 and α‐SMA expression were quantified in liver biopsies collected from NASH patients (n = 26) with different fibrosis stages and PNPLA3 genotypes. To study the potential mechanisms driving PNPLA3 expression during NASH progression towards fibrosis, hepatocytes and hepatic stellate cells (HSCs) were cultivated in low and high glucose medium. Moreover, hepatocytes were treated with increasing concentrations of palmitic acid alone or in combination with glucose. Conditioned media were collected from challenged hepatocytes to stimulate HSCs.

**Results:**

Tissue expression of PNPLA3 was significantly enhanced in biopsies of patients carrying the I148M polymorphism compared to wild type (WT). In NASH biopsies, PNPLA3 significantly correlated with fibrosis stage and α‐SMA levels independently of PNPLA3 genotype. In line, PNPLA3 expression was higher in α‐SMA positive cells. Low glucose increased PNPLA3 in HSCs, whereas high glucose induced PNPLA3 and de‐novo lipogenesis‐related genes expression in hepatocytes. Palmitic acid induced fat accumulation and cell stress markers in hepatocytes, which could be counteracted by oleic acid. Conditioned media collected from lipotoxic challenged hepatocytes markedly induced PNPLA3 mRNA and protein levels, fibrogenic and autophagic markers and promoted migration in HSCs. Notably, conditioned media collected from hepatocytes cultivated with both glucose and palmitic acid exacerbated HSCs migration, PNPLA3 and fibrogenic gene expression, promoting release of cytokines from HSCs.

**Conclusions:**

Collectively, our observations uncover the diverse metabolic regulation of PNPLA3 among different hepatic cell populations and support its relation to fibrosis progression.

**Abbreviations**
ACC1: acetyl‐CoA carboxylase 1
ALT: alanine transaminase
AST: aspartate transaminase
c.m.: conditioned medium
CEBPA: Ccaat/Enhancer‐Binding Protein, alpha
ChREBP: carbohydrate responsive element‐binding protein
ECM: extracellular matrix
ER stress: endoplasmic reticulum stress
FASN: fatty acid synthase
FFA: free fatty acid
Glc: glucose
GM‐CSF: granulocyte‐macrophage colony‐stimulating factor
GRP78: glucose regulated protein 78KD
HCC: hepatocellular carcinoma
HSCs: hepatic stellate cells
JNK: c‐Jun N‐terminal kinase
MIF: macrophage migration inhibitory factor
NAFLD: non‐alcoholic fatty liver disease
NASH: non‐alcoholic steatohepatitis
PI3KIP1: phosphoinositide 3 kinase interacting protein 1
p‐JNK: phosphorylated JNK
PNPLA3 I148M: patatin‐like phospholipase domain‐containing protein 3 genetic variant I148M (G/G)
SCD‐1: Stearoyl‐CoA desaturase 1
SREBP‐1c: sterol regulatory element‐binding protein 1c
TG: triglycerides
WT PNPLA3: patatin‐like phospholipase domain containing protein 3 wild type (C/C)
α‐SMA: α‐smooth muscle actin

**Lay summary**

Hepatic PNPLA3 expression increases during progression of liver fibrosis in patients with NASH and mainly colocalizes with activated hepatic stellate cell marker α‐SMA. High glucose and saturated free fatty acids increase PNPLA3 and induce lipid stress in hepatocytes, which in turn results in PNPLA3 induction in hepatic stellate cells along with exacerbated pro‐fibrogenic features.

## 1 INTRODUCTION

Non‐alcoholic fatty liver disease (NAFLD) is a growing and burgeoning health issue with high prevalence in western countries.^1^, ^2^ A portion of NAFLD patients (approximately 5%‐10%) evolves into non‐alcoholic steatohepatitis (NASH), which represents the advanced manifestation of NAFLD and predisposes to severe hepatic impairment, such as cirrhosis and ultimately cancer.^3^ Current evidence suggests that the progression from steatosis towards NASH is a multi‐hit process triggered by sustained hepatocytes injury resulting in inflammation and fibrosis.^4^ A recognized hallmark of this process is the accumulation of excessive free fatty acids (FFAs) in hepatocytes stored as triglycerides (TGs) in lipid droplets (LDs). The source of this FFAs flux towards hepatocytes derives from adipose tissue lipolysis, increased hepatic de novo lipogenesis and external factors such as imbalanced diets rich in sugars and saturated FFAs (i.e. western diet). In particular, saturated fatty acids, like palmitic acid (PA; C16:0), are known to be lipotoxic and promote metabolic stress and apoptosis in hepatocytes.^5^, ^6^, ^7^ The consequence of sustained hepatocellular injury is the activation of hepatic stellate cells (HSCs), the main contributors in driving liver fibrosis as wound healing response, characterized by the replacement of normal hepatic parenchyma with fibrotic scars.^8^, ^9^ Liver fibrosis is considered the major determinant of mortality in patients with NASH.^10^ Emerging evidence indicates that also genetic factors play a key role in hepatic disease progression and fibrosis development.^11^ In this regard a single base polymorphism of the human patatin‐like phospholipase domain‐containing 3 (PNPLA3) gene, known as I148M (rs738409 C>G) has been linked to increased risk of hepatic TG accumulation^12^, ^13^ and is considered as an independent risk factor predisposing to NASH.^14^, ^15^, ^16^, ^17^ In the liver, PNPLA3 expression is regulated by carbohydrate feeding via sterol regulatory element‐binding protein‐1c (SREBP‐1c) in mouse and human hepatocytes.^18^, ^19^, ^20^ Notably, PNPLA3 is highly expressed in HSCs^21^ and recent findings uncovered a strong contribution of PNPLA3 to achieve a full myofibroblast‐like phenotype in HSCs and recognized a significant impact of its genetic variant I148M to exacerbate pro‐inflammatory and pro‐fibrogenic features of human HSCs.^22^, ^23^ However, the regulation of PNPLA3 during human hepatic fibrosis development is largely unclear. Therefore, we aimed to investigate whether: (a) hepatic PNPLA3 expression correlates with fibrosis progression in human NASH and according to the I148M genotype; (b) PNPLA3 expression is regulated by glucose and FFAs in hepatocytes as well as in HSCs; (c) metabolic stress in hepatocytes affects PNPLA3 expression and fibrogenic features in HSCs.

## 2 MATERIALS AND METHODS

### 2.1 Human samples

Liver biopsy specimens (n = 26) were collected from individuals with elevated transaminase values after routine examination with suspected NAFLD/NASH. Patients gave informed consent at the time of recruitment and their records were anonymized and de‐identified. PNPLA3 genotyping, patient's data and pathologic evaluations were approved by Ethics Committee of the Medical University of Vienna (EC: 2032/2013 and 1235/15, respectively). All the samples were obtained and categorized by a pathologist (MH) from Medical University of Vienna. Steatosis percentage (%), inflammation, hepatocellular ballooning and NAS score were evaluated. Cases with NAS score ≥3 and fibrosis stage from 1 to 4 were classified as NASH. According to the NASH Clinical Research network guidelines, fibrosis stages were classified as follows: F0 = none; F1 = perisinusoidal or periportal; F1a = mild, zone 3, perisinusoidal; F1b = moderate, zone 3, perisinusoidal; F1c = peri‐portal sinusoidal fibrosis; F2 = Zone 3 sinusoidal fibrosis and peri‐portal sinusoidal fibrosis; F3 = Bridging fibrosis; F4 = Cirrhosis.^24^ For convenience, from 1 to 2 were considered as ‘mild’ fibrosis, whereas fibrosis stage from 3 to 4 as ‘severe’ fibrosis. PNPLA3 genotyping was performed by real‐time PCR for the I148M single‐nucleotide polymorphism (rs738409 C>G), as done routinely in our clinical research center.^22^, ^23^ Only homozygote genotypes were used in this study (C/C as WT and G/G as I148M).

### 2.2 Human tissue staining and image quantification

For immunofluorescence (IF) staining, slides were blocked for 1 hour, incubated overnight at 4°C with polyclonal rabbit anti‐human PNPLA3 antibody (#ab81874, Abcam). The day after, slides were washed and incubated at room temperature for 1 hour with monoclonal mouse anti‐human α‐SMA antibody (#A5228, Sigma‐Aldrich). Slides were then washed and incubated with the respective secondary antibody: Alexa Fluor 488 goat anti mouse IgG for α‐SMA and Alexa Fluor 594 goat anti rabbit IgG for PNPLA3 (both Thermo Fisher Scientific). Nuclei were counterstained with DAPI (Sigma‐Aldrich) and mounted (VECTASHIELD® Mounting Medium, Vector Laboratories) for microscope analysis (Olympus BX51). Relative quantification of IF staining was performed using Image J software in an automated fashion as seen previously.^25^ Channels were splitted for the respective fluorochrome (Red for PNPLA3 and Green for α‐SMA), and total fluorescence was quantified using macros in parallel for all the pictures taken. A number of six random shots were taken for each slide and quantified accordingly.

### 2.3 Cell culture

Immortalized human hepatocytes (IHH),^26^ hepatocellular carcinoma cell line HuH7 (ATCC) and immortalized hepatic stellate cell line LX‐2 (kindly provided by Prof. S. L. Friedman, Mount Sinai School of Medicine), were cultivated in Dulbecco's modified Eagle's medium (DMEM) with 4.5 g/L glucose (Thermo Fisher Scientific) supplemented with 10% heat inactivated FBS (IHH and HuH7) or 5% non‐heat inactivated FBS (LX‐2), L‐glutamine (2 mmol/L) and antibiotics (all from Sigma‐Aldrich).^22^ The genotype of each cell line used in this work has been analyzed by real‐time PCR for the I148M SNP, as done routinely in our clinical research center. IHH, HuH7 and LX‐2 expressed the 148M PNPLA3. For glucose (Glc) experiments, cells were starved for 5 hours in glucose‐free medium prior treatment using high (25 mmol/L) or low Glc (5 mmol/L) for additional 24 hours. For FFA experiments, cells were seeded overnight in serum‐free medium and then cultivated in medium without FFA or in medium with palmitic acid (PA) alone or in combination with oleic acid (OA) at different concentrations for 24 hours. 5 mmol/L stock solutions of PA and OA were prepared by dissolving the substances in a mixture of 0.01 mol/L NaOH (25%, v/v) and 150 mmol/L NaCl (40%, v/v) while heating. After heating fatty acid free BSA solution (24%, v/v in 150 mmol/L NaCl) was added to reach 18% v/v of the total concentration. PBS (10%, v/v) was added and filled up with distilled sterile water to 100%. For experiments using conditioned media (c.m.) collected from hepatocytes to stimulate HSCs for 24 hours, we used the following conditions: (a) culture media from hepatocytes (HuH7/IHH) without FFA for 24 hours (c.m. no FFA); (b) culture media from hepatocytes treated with PA alone or in combination with OA for 24 hours (c.m. PA; c.m. PA + OA); (c) culture media from hepatocytes treated with low or high Glc alone or (d) in combination with PA.

#### 2.3.1 Luciferase assay

Luciferase assay was performed as previously reported.^22^, ^23^ Briefly, IHH and LX‐2 cells were seeded in a 24‐well plate and transiently transfected with a DR4‐TK‐luc (LXRE a generous gift of Dr David Mangelsdorf, UT Southwestern) or with a SRE‐TK‐pGL3 (containing an E‐box cloned in 3 copies in the SmaI site of the TK‐pGL3) for SREBP‐1c together with the pcDNA3.1‐2xFLAG‐SREBP1c a generous gift of Dr Timothy Osborne (Addgene plasmid # 26802; http://n2t.net/addgene:26802; RRID: Addgene_26802) or the pSV Sport SREBP1c dominant negative a gift from Dr Bruce Spiegelman (Addgene plasmid # 8885; http://n2t.net/addgene:8885; RRID: Addgene_8885). After 24 hours the medium was replaced with DMEM glucose free and cells were treated as previously described for additional 24 hours. Cells were lysed using a solution (4% Triton‐X100, Glycyl‐Glycine 100 mmol/L, MgSO4 100 mmol/L, EGTA 250 mmol/L) for 1 hour at room temperature on a shaker platform. The lysates were then combined with the substrate solution (Luciferin 2.5 mmol/L and ATP 20 mmol/L, Sigma‐Aldrich) and analyzed with a luminometer (Lumat LB9507; EG&G Berthold).^27^

### 2.4 RT‐PCR analysis

RNA was isolated from primary HSCs and LX‐2 by NucleoSpin® RNA (MACHEREY‐NAGEL GmbH & Co.) according to manufacturer's instructions. One µg of RNA was reverse transcribed using Moloney Murine Leukemia Virus enzyme, 10xbuffer for cDNA synthesis and Deoxynucleotide Mix 10 mmol/L (Sigma‐Aldrich). Two microliters of diluted cDNA were loaded together with SYBR® Select Master Mix on 96‐well plate to perform RT‐PCR analysis (Thermo Fisher Scientific). Forward and reverse primers for each gene of interest were specifically designed and used at 10 pmol/µL (Eurofins Genomics). Sequences are available upon request.

### 2.5 Western blot

Protein expression analysis was performed as described previously.^22^, ^23^

### 2.6 Migration assay

Cell migration was measured in modified Boyden chambers, as described previously.^22^, ^23^ Cells were fixed in methanol and stained with hematoxylin/eosin solution before counting.

### 2.7 Elisa cytokine assay

HSCs supernatant was collected after 24 hours of exposure to IHH c.m. pre‐incubated with 25 mmol/L Glc alone or in combination with PA (300 μmol/L). Proteome profiler for human cytokine array (R&D) was performed according to manufacturer instructions, as previously described.^22^

### 2.8 Flow cytometry

LX‐2 and IHH were previously treated with 5 or 25 mmol/L of Glc, trypsinized and resuspended in fixing and permeabilizing reagents (all Thermo Fisher Scientific). Thereafter, they were incubated with a 1:500 dilution of the polyclonal rabbit anti human PNPLA3 antibody (Abcam) for 1 hour, on a shaker platform. A fluorochrome‐labelled Alexa Fluor 594 goat anti rabbit IgG was incubated for 1 hour at room temperature on a shaker, DAPI was added to each tube for 10 minutes before flow cytometric analysis. Flow cytometry was performed with BD FACSCanto™ II and BD FACSDiva™ software (Becton Dickinson).

### 2.9 Statistics

Data are presented as mean values ± standard deviation (SD). Correlations were analyzed non parametrically by Spearman's rank correlation. Statistical analysis was performed using GraphPad Prism. The unpaired Student *t* test was used when two groups were compared, and two‐way analysis of variance was used for multiple groups. *P* < .05 was considered statistically significant.

## 3 RESULTS

### 3.1 PNPLA3 correlated with fibrosis development in human NASH and is more expressed in α‐SMA positive cells

Based on our previous observation that PNPLA3 expression was induced during primary human HSCs activation in vitro,^22^ we evaluated PNPLA3 expression during liver fibrosis development in human NASH biopsies. Our study population involved 26 individuals (Table 1) and was classified according to PNPLA3 genotype (two groups, C/C – WT, n = 15 and G/G ‐ I148M, n = 11). No significant differences were observed in clinical aspects between these two groups, apart from a trend in increased transaminases level of ALT and AST in carriers of the genetic variant I48M (G/G), compatible with previous reports involving larger sample size.^13^, ^14^

**Table 1:** Demographic and clinical data of patient cohort (n = 26)

	C/C (n = 15)	G/G (n = 11)
Age (y)	47.9 ± 12.2	52.3 ± 14.3
Sex (m:w)	8:7	6:5
BW (kg)	98.2 ± 25.2	86.9 ± 21.2
Size (cm)	171.1 ± 9.9	171.7 ± 9.8
BMI	33.5 ± 7.4	29.2 ± 4.8
ALT (U/L)	66.5 ± 31.4	96.1 ± 64.8
AST (U/L)	54.4 ± 42.6	75.6 ± 75.1
ALP (U/L)	115.7 ± 92.4	80.4 ± 19.7
GGT (U/L)	287.6 ± 437.5	104.1 ± 89.6
Ferritin (µg/L)	221.1 ± 258.7	211.9 ± 204.4
TG (mg/dL)	174.7 ± 106.2	152.6 ± 78.5
T‐Chol (mg/dL)	193.6 ± 53.5	177.6 ± 32.7
HbA1c (%)	5.7 ± 0.9	5.8 ± 1.0
Statin [%]	20.0	18.2
Diabetes [%]	40.0	36.6

Note

Demographic and clinical features of patients underwent percutaneous liver biopsy for suspected NAFLD/NASH. Data are expressed as mean values ± SD.

In addition to PNPLA3 genotype, the cohort of liver samples was divided and analyzed according to fibrosis stage, ranging from F = 1 and 2 for ‘mild’/periportal to F = 3 and 4 for ‘severe’ hepatic fibrosis/cirrhosis (Table 2). Notwithstanding the low sample size, significantly higher steatosis percentage (%) was found in G/G genotypes independently of the fibrosis stage (Table 2).

**Table 2:** Key histopathological data

	F1‐F2		F3‐F4	
	C/C	G/G	C/C	G/G
Steatosis (%)	55 ± 12	72.6 ± 11*	32.5 ± 14	49.3 ± 15.2*
Inflammation	1.1 ± 0.2	1.6 ± 0.1	1 ± 0.3	1.3 ± 0.2
Ballooning	1.4 ± 0.6	1.6 ± 0.6	1.5 ± 0.7	1.7 ± 0.2
NAS	4 ± 0.6	5.25 ± 0.8	3.5 ± 1.2	4.1 ± 0.5

Note

Pathological characteristics of the cohort of patients assessed by board‐certified pathologist. Data are presented as mean values ± SD.

* *P* < .05 vs C/C genotype (PNPLA3 WT = C/C; PNPLA3 I148M = G/G).

**Figure 1:**
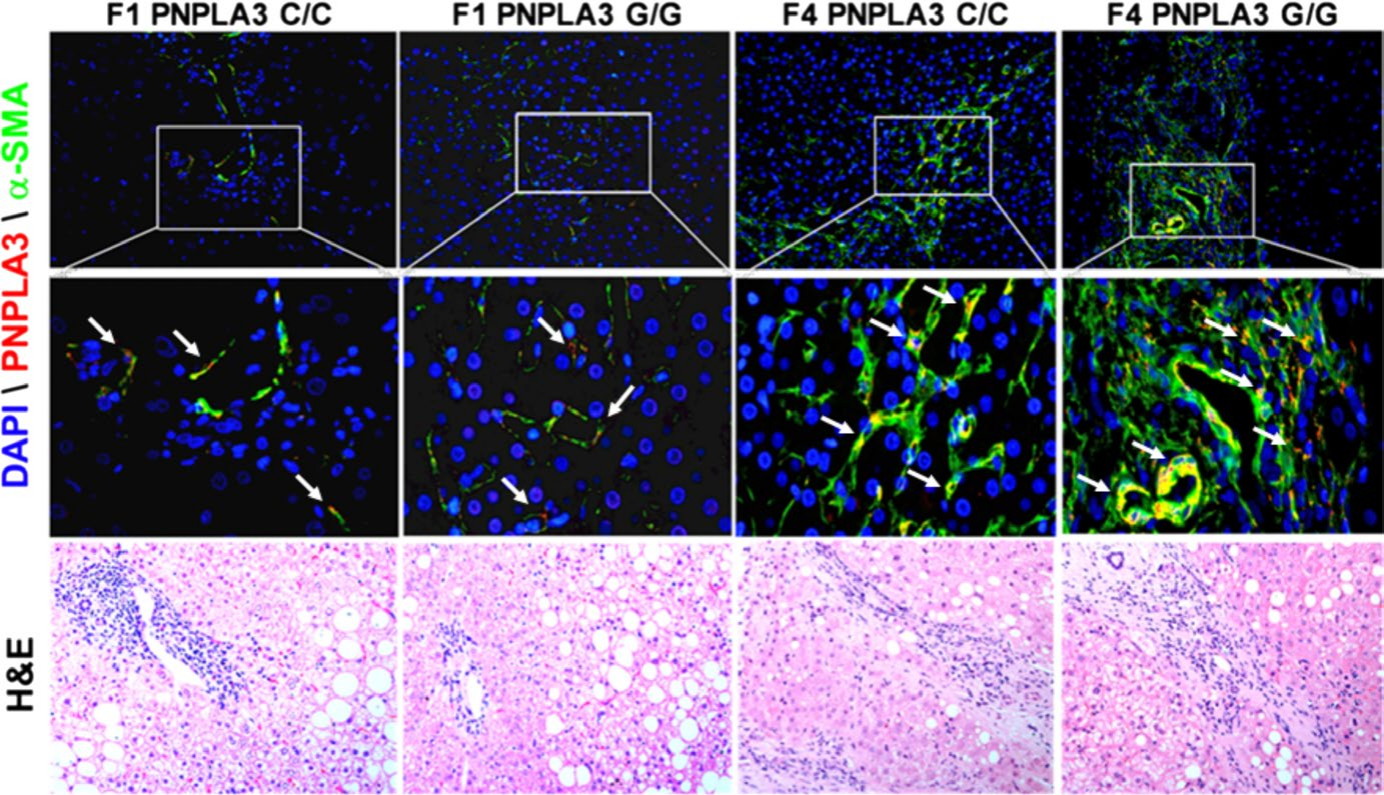
PNPLA3 increases during liver fibrosis development in patients with NASH. Immunofluorescence (IF) images (n = 6 random images per tissue slide) performed on liver biopsies from NASH patients (n = 26) grouped as described in Materials and Methods and in Tables 1 and 2. DAPI was used to counterstain the nuclei. Please note that HE staining does no directly correspond to the areas shown in the IF sections. PNPLA3 = red channel; α‐SMA = green; DAPI = blue. White arrows indicate cells positive for both PNPLA3 and α‐SMA (yellow)

**Figure 2:**
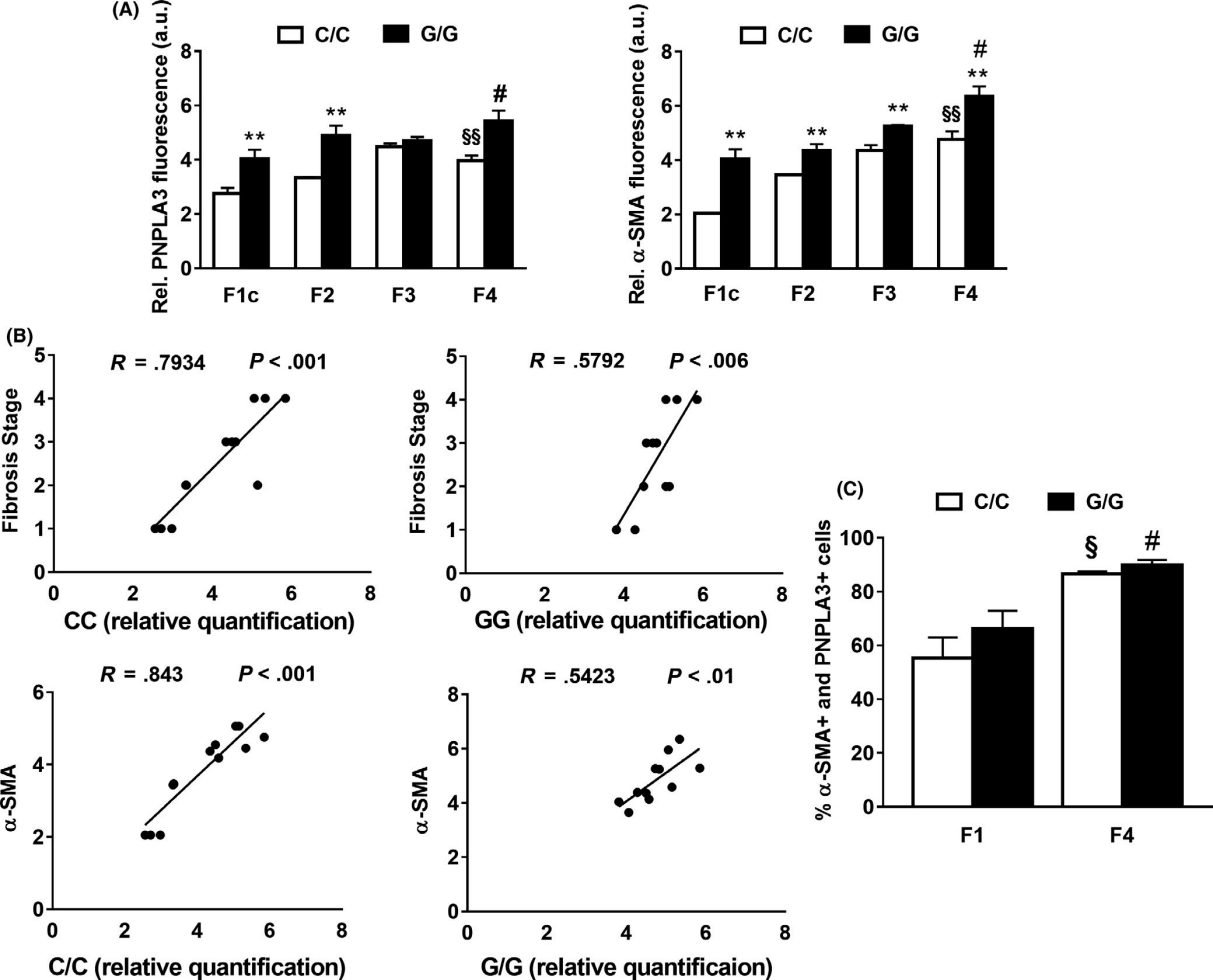
PNPLA3 correlates significantly with the pro‐fibrogenic marker α‐SMA and with the fibrosis stage in NASH patients. A, Quantitative relative fluorescence amount for PNPLA3 (red) and α‐SMA (green) calculated from pictures (randomly chosen as n = 6 per liver slide) using ImageJ software and obtained from human liver biopsies, carrying the WT PNPLA3 with different fibrosis stage (F1‐F2‐F3‐F4; a.u. = arbitral units). **P < .01 F1c C/C vs G/G; §*P* < .05 F1c C/C vs F4 C/C; §§ *P* < .01 F1c C/C vs F4 C/C; #*P* < .05 F4 C/C vs G/G. B, Correlation between PNPLA3 relative quantification and fibrosis stage(R = 0.579, *P* = .006) with PNPLA3 in both PNPLA3 genotypes. Correlation between PNPLA3 and α‐SMA tissue relative quantification. (C/C: R = 0.843, *P* < .001; G/G: R = 0.5423, *P* < .01). Data shown as mean values ± SD. C, Colocalization expressed as hepatic cells percentage positive for both PNPLA3 and α‐SMA (% of cells positive for PNPLA3 and α‐SMA), during progression of liver fibrosis from F1 to F4, in C/C and G/G carriers. Analysis performed using ImageJ software (n = 4 each fibrosis stage and PNPLA3 genotype). §*P* < .05 F1c C/C vs F4 C/C; #*P* < .05 F4 C/C vs F4 G/G. Data shown as mean values ± SD. White bars stay for C/C PNPLA3 and black bars stay for G/G PNPLA3 throughout the figure panels. F1c = peri‐portal sinusoidal fibrosis; F2 = Zone 3 sinusoidal fibrosis and peri‐portal sinusoidal fibrosis; F3 = Bridging fibrosis; F4 = Cirrhosis^24^

PNPLA3 and α‐SMA tissue expression were investigated in these samples by immunofluorescence (IF) staining (Figure 1 and Supp. Figure S1). Strikingly, we found that PNPLA3 expression increased during the progression of liver disease from mild to severe fibrosis (Figure 2A), with higher amount for the G/G variant compared to the C/C (Figure 2A, F1: C/C vs G/G from 2.7 ± 0.2 to 4.04 ± 0.32, *P* < .01; F4: C/C vs G/G from 3.9 ± 0.18 to 5.4 ± 0.39; *P* < .05). Importantly, relative quantification of the activated HSCs marker α‐SMA significantly increased in patients with the G/G genotype compared to the C/C carriers during fibrosis development, as PNPLA3 (Figure 2A). These observations confirmed and supported our previous findings that PNPLA3 is proportionally induced during human NASH progression.^22^

Moreover, PNPLA3 relative quantification correlated significantly with the fibrosis stage (R = 0.793, *P* < .001 for C/C and R = 0.579, *P* = .006 for PNPLA3 G/G) and with α‐SMA tissue expression independently of the genotype (R = 0.843, *P* < .01 for C/C and R = 0.5423, *P* < .01 for G/G) (Figure 2B).

As previously reported, PNPLA3 is known to be expressed in hepatocytes and HSCs.^21^, ^22^ Notably, our immunofluorescence staining analysis revealed that during fibrosis progression PNPLA3 liver expression increases mainly in cells positive for the HSCs activation marker α‐SMA (Figure 2C), along with lower visible hepatocellular staining.

Collectively, our data clearly report that hepatic PNPLA3 expression is significantly induced during liver fibrosis development and it is more abundant in carriers of G/G genotype. Importantly, hepatic PNPLA3 level correlate both with fibrosis stage and relative quantification of α‐SMA, independently of the genotype. In addition, most of PNPLA3 tissue expression localizes in cells positive for the activated HSCs marker α‐SMA.

### 3.2 PNPLA3 is oppositely regulated by glucose in human hepatocytes and HSCs

**Figure 3:**
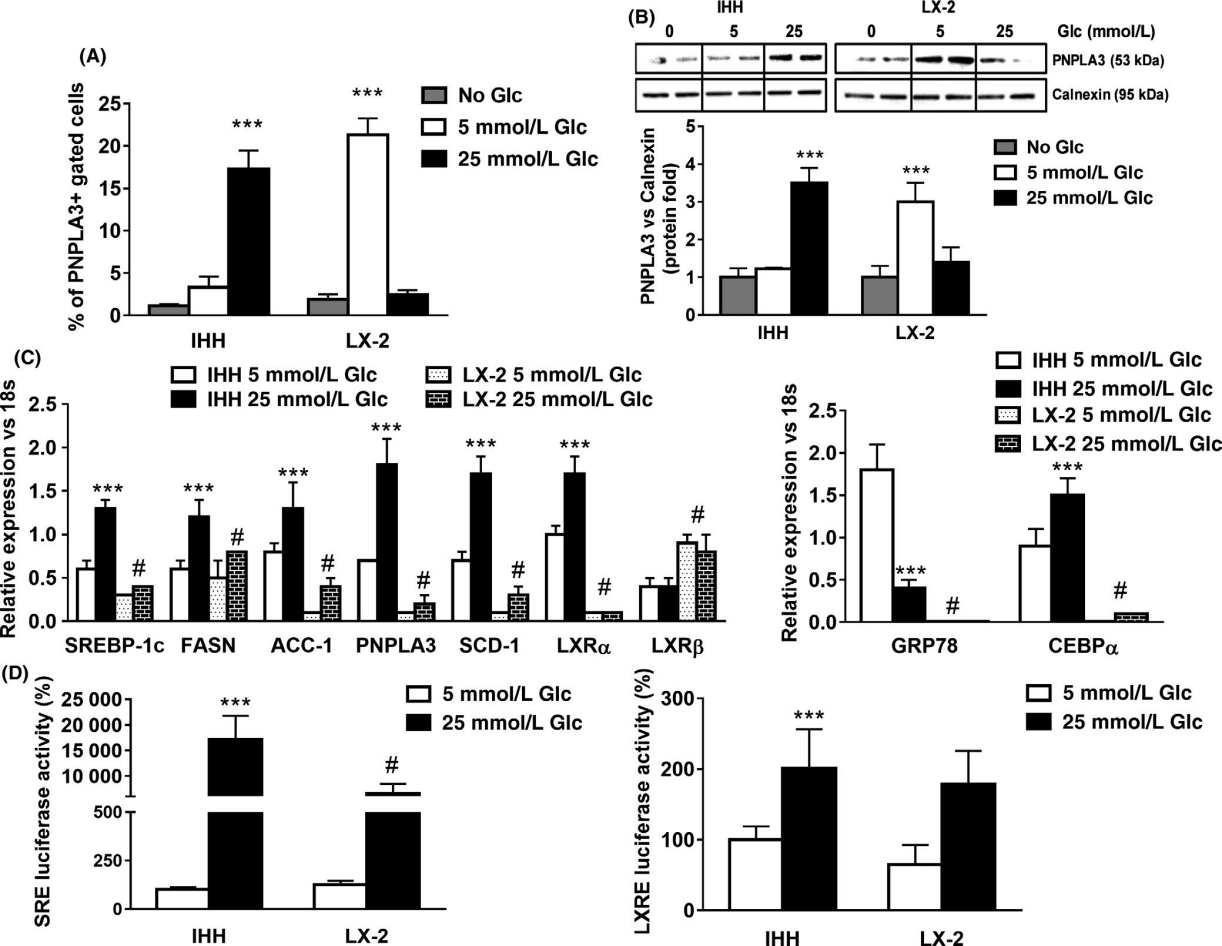
High glucose (25 mmol/L) induces PNPLA3 and de‐novo lipogenic genes expression in hepatocytes, but not in HSC. LX‐2 and IHH cells were cultured in medium without glucose (No Glc, grey bars) or in medium containing low (5 mmol/L, white bars) or high (25 mmol/L, black bars) glucose (Glc) for 24 h. A, PNPLA3 intracellular staining was analyzed by flow cytometry. Data displayed as percentage (%) of gated PNPLA3 positive cells. ***P < .001 vs No Glc (grey bars). B, Representative blots of protein extracts collected from IHH and LX‐2. Depicted protein expression of PNPLA3 was calculated using ImageJ software. Calnexin was used as a loading control. ***P < .001 vs No Glc. C, Expression of SREBP‐1c, FASN, ACC‐1, PNPLA3, GRP78, CEBPα, SCD‐1, LXRα and β from IHH and LX‐2 cells analyzed by real‐time PCR. Data normalized to 18s. ***P < .001 vs IHH 5 mmol/L Glc, #*P* < .001 vs IHH 25 mmol/L Glc. D, Luciferase activity of IHH and LX‐2 cells transiently transfected with either SREBP‐1c response element (SRE) plasmids or LXR response element (LXRE). All data displayed represents three independent experiments performed in duplicates. Data shown as mean values ± SD. ***P < .001 vs IHH 5 mmol/L Glc, #*P* < .001 vs IHH 25 mmol/L Glc

To unravel the potential mechanisms determining the induction of PNPLA3 and to mimic the starving conditions of patients undergoing liver biopsy, we next measured PNPLA3 expression in LX‐2 and IHH, human HSCs and hepatocyte cell lines respectively, using culture media with low (5 mmol/L) or high (25 mmol/L) glucose (Glc). Firstly, flow cytometry analysis showed that the amount of PNPLA3 was significantly lower in hepatocytes compared to HSCs (*P* < .001) under low Glc conditions (5 mmol/L); when cells were treated with high Glc (25 mmol/L), the response was reversed (*P* < .001, Figure 3A). These data were confirmed by western blot analysis (Figure 3B). Secondly, sterol regulatory element‐binding protein‐1c (SREBP‐1c) expression and its downstream targets coding for de‐novo lipogenesis pathway (FASN, ACC‐1, SCD‐1 and PNPLA3) were significantly induced by 25 mmol/L Glc in IHH (*P* < .001, Figure 3C), but blunted in HSCs.

Downstream of SREBP‐1c,^28^ the expression of liver X receptor α (LXRα) was also upregulated upon 25 mmol/L Glc in IHH whereas there was no change in HSCs. Inversely, the β isotype (LXRβ) expression increased after high Glc treatment in LX‐2, but not in hepatocytes (*P* < .001, Figure 3C), suggesting differences in pathways controlling lipid/cholesterol levels between hepatocytes and HSCs. Interestingly, the expression of the CCAAT/Enhancer‐Binding Protein alpha (CEBPα), a transcription factor known to control hepatic gluconeogenesis/de‐novo lipogenesis,^29^, ^30^ was strongly induced by high Glc in hepatocytes. Furthermore, the expression of the glucose regulated protein 78KD (GRP78) showed opposite trends (*P* < .001, Figure 3C), confirming the dual role of this protein in inhibiting SREBP‐1c‐induced de‐novo lipogenesis.^31^, ^32^

To support mRNA gene expression data, we performed luciferase co‐transfection assays using LXR (LXRE) and SREBP‐1c (SRE) response elements in IHH and LX‐2 followed by Glc stimulation (5 or 25 mmol/L). Indeed, high Glc dose stimulated significantly SREBP‐1c transcriptional activity in IHH, compared to LX‐2 cells (Figure 3D, *P* < .001).

Collectively, our data suggest that PNPLA3 transcript and protein levels are induced by high glucose (25 mmol/L) in human hepatocytes, along with de‐novo lipogenesis genes expression. Conversely, in LX‐2 PNPLA3 protein levels are higher when glucose levels are low (5 mmol/L).

### 3.3 FA loading induces metabolic stress in hepatocytes which results in increased PNPLA3, pro‐fibrogenic genes and autophagy in HSCs

**Figure 4:**
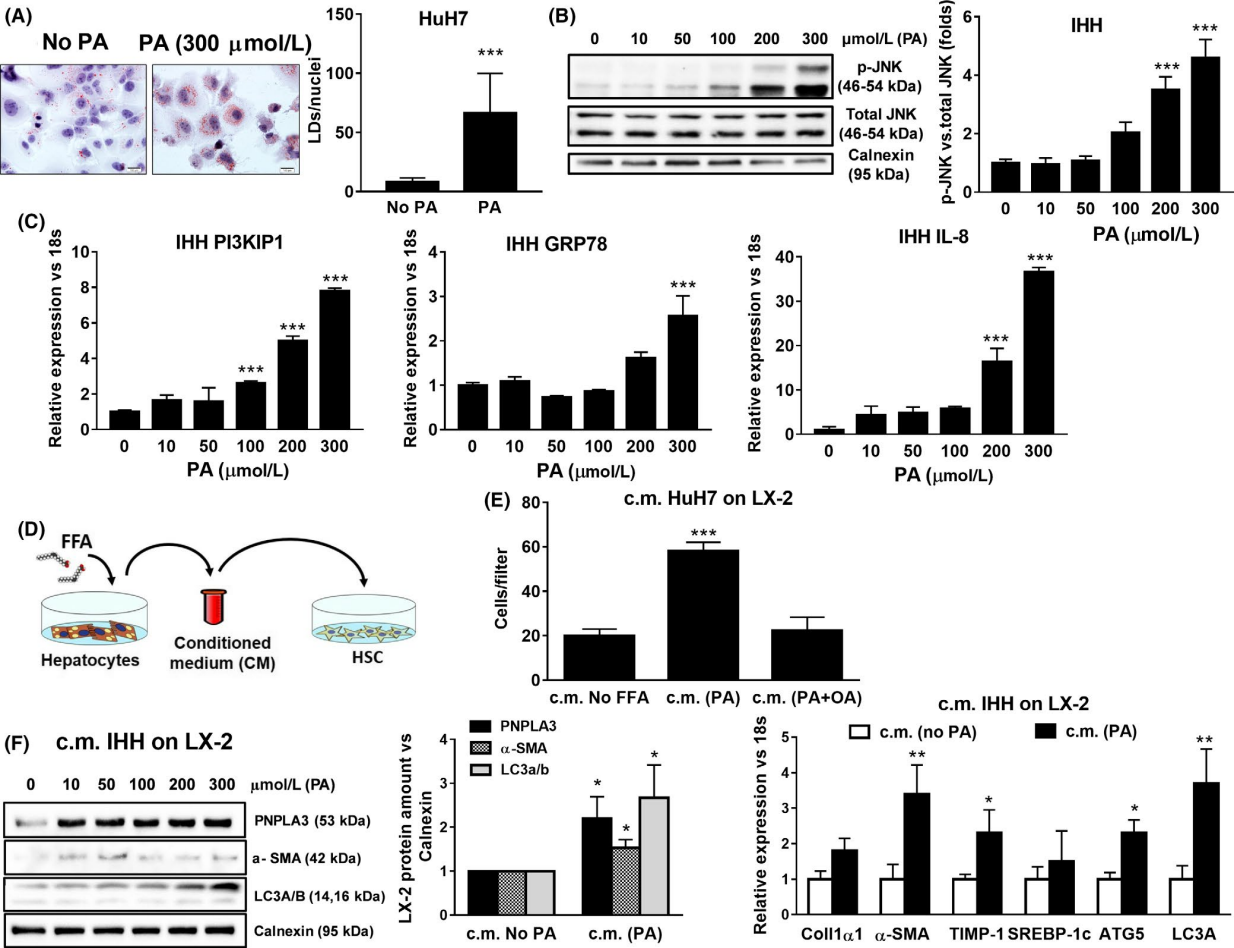
Conditioned medium (c.m.) form lipid stressed hepatocytes induced migration, PNPLA3, pro‐fibrogenic and autophagic markers expression in HSCs. Hepatoma cell lines (HuH7) and immortalized human hepatocytes (IHH) were cultivated without (No PA) or with 300 μmol/L of palmitic acid (PA) for 24 h to induce lipid accumulation and cellular stress. A, Oil Red O staining was performed on fixed HuH7 cells after 300 μmol/L PA treatment, as described in Materials and Methods. Lipid droplets (LDs) amount was calculated using BioPix iQ Imaging Software and normalized on the number of nuclei (LDs/nuclei). ***P < .001 vs No PA. B, Representative blots of phospho‐JNK (p‐JNK) performed on protein extracts collected from IHH cells. Densitometry analysis calculated using ImageJ software and data normalized to total JNK. ***P < .001 vs IHH without PA. C, Expression of PI3KIP1, GRP78 and IL‐8 in IHH analyzed by real‐time PCR and normalized to 18s. Data displayed represents three independent experiments performed in duplicates. ***P < .001 vs IHH without PA. D, Schematic representation of conditioned medium (c.m.) collected from hepatocytes (IHH and HuH7) cultivated for 24 h without FFA (c.m. No FFA) or with different concentrations of PA (up to 300 μmol/L) or in combination with oleic acid (OA; ratio 1:2) and then used to stimulate untreated HSCs, for additional 24 h. E, Migration was evaluated in LX‐2 using modified Boyden chambers for 6 h, after exposure to c.m. from lipid‐laden HuH7 cells treated as indicated in bar graph. Data are presented as number of cells per filter migrated towards the lower chamber. Data shown are representative of three independent migration assays. ***P < .001 vs c.m. HuH7 no FFA. (F, left panel) C.m. collected from IHH cultivated for 24 h without PA (c.m. No PA) or with PA at different concentrations and then used to stimulate LX‐2 for 24 h. Depicted PNPLA3, α‐SMA, LC3 A/B protein expression was analyzed by western blotting and data normalized to Calnexin. *P < .05 vs c.m. No FFA. (F, right panel) Expression of Collagen 1α1, α‐SMA, TIMP‐1, SREBP‐1c, ATG5 and LC3a analyzed by real‐time PCR and normalized to 18 s. Data displayed represents three independent experiments performed in duplicates. Data shown as mean values ± SD. *P < .05 and **P < .01 vs c.m. No PA

Lipotoxic saturated FFAs impair lipid accumulation in hepatocytes and contribute actively to inflammation and activation of HSCs.^33^, ^34^ In order to create an in vitro model resembling lipid‐stressed hepatocytes,^33^ human immortalized hepatocytes and hepatoma cell lines (IHH and HuH7, respectively) were challenged with increasing concentrations of palmitic acid (PA) alone or in combination with oleic acid (OA). After 24 hours, fatty acid loading was confirmed by Oil Red O staining and quantified using BioPix iQ Imaging Software (*P* < .05, Figure 4A). To confirm palmitate‐induced lipotoxicity in hepatocytes,^33^, ^34^ we performed western blot analysis and observed that highest phosphorylation of c‐Jun N‐terminal kinases (JNK) was at PA concentration of 300 μmol/L (*P* < .001, Figure 4B). PA treatment also induced expression of ER stress markers, such as phosphoinositide‐3‐kinase‐interacting protein 1 (PI3KIP1),^7^ GRP78 and interleukin‐8 (IL‐8) (Figure 4C),^35^ while the combination with OA (PA:OA, 1:2 ratio) prevented the above reported induction of PI3KIP1, IL‐8 and activation of JNK pathway, thus protecting hepatocytes from lipotoxic stress signals (*P* < .01 and *P* < .001, Figure S2A,B).

Next, to mimic cell interactions in vivo, we investigated how lipid‐stressed hepatocytes affected fibrogenic response in HSCs in vitro.

Interestingly, conditioned media (c.m.) collected from hepatocytes cultivated with only PA‐induced migration in HSCs, whereas the combination with OA prevented this effect (Figure 4D,E; Figure S2 and S3). In line with migration, PNPLA3, α‐SMA and LC3A/B (autophagy marker) protein levels were also significantly increased, starting off at low concentrations (ie 50µM PA; Figure 4F). Inversely, c.m. collected from hepatocytes treated with the combination of PA and OA was protective^36^ and prevented these effects in HSCs (Supporting Figure S2 and S3). In addition, HSCs fibrogenic markers Collagen1α1, α‐SMA and TIMP‐1 were induced, along with autophagy markers ATG5 and LC3a, thus contributing to sustain HSCs activation (Figure 4F).

Altogether, these data evidenced that excessive exposure to lipotoxic PA in hepatocytes activates HSCs, thus resulting in upregulation of PNPLA3, pro‐fibrogenic markers as well as autophagy and migration in HSCs.

### 3.4 Hepatocytes conditioned medium containing glucose in combination with PA exacerbated pro‐fibrogenic features of HSCs

**Figure 5:**
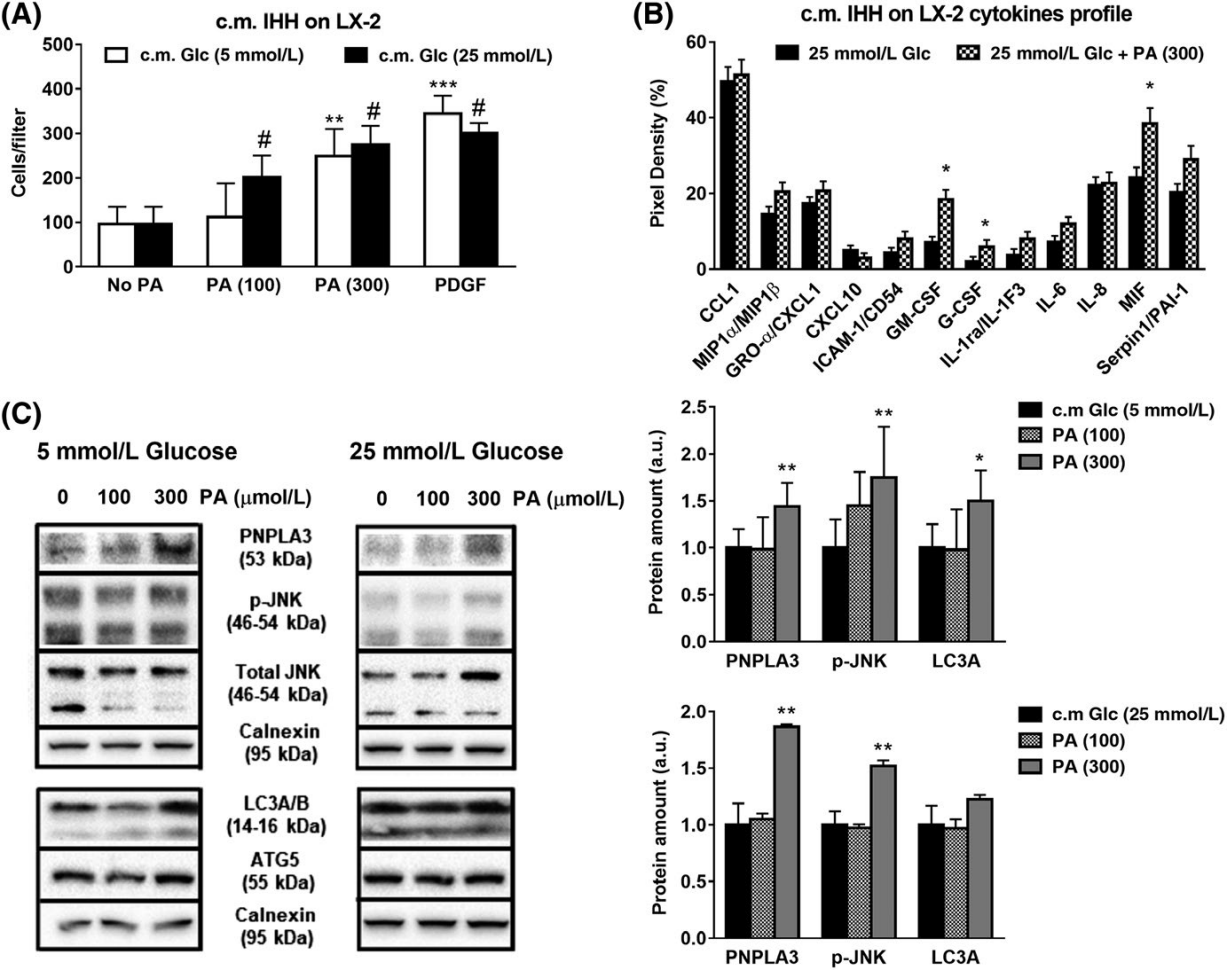
Conditioned medium (c.m.) collected from hepatocytes cultivated with PA and high glucose increases migration, pro‐fibrogenic and autophagy markers and cytokines release in HSCs. IHH cells cultivated with low (white bars) or high Glc (black bars) alone or in combination with PA (100 and 300 μmol/L). After 24 h, conditioned medium (c.m.) was collected and used to stimulate HSCs for 24 h. Conditions used in IHH cells are described in Material and Methods. A, Migration assay was performed in LX‐2 using Boyden chambers, as described above. PDGF (10 ng/ml) was used as positive control. **P < .01 and ***P < .001 vs c.m. No PA, 5 mmol/L Glc (white bar), #*P* < .01 vs No PA, 25 mmol/L Glc (black bar). B, Cytokine array quantification plot displayed and performed in media collected from LX‐2 previously exposed to IHH c.m., as described in Materials and Methods. *P < .05 vs 25 mmol/L Glc (black bars). C, Depicted expression of PNPLA3, phospho‐JNK (p‐JNK), total JNK, LC3A/B and ATG5 analyzed by western blotting. Calnexin used as loading control (protein amount reported as arbitrary units = a.u.). To evaluate pathway activity, p‐JNK measured in comparison with total JNK. Representative blot displayed. Densitometry analysis was calculated using ImageJ software. *P < .05, **P < .01 vs c.m. 5 or 25 mmol/L Glc (black bars)

The above observations brought us to verify whether the combination of both Glc and PA might exacerbate these fibrogenic features in HSCs. Therefore, we challenged IHH cells with PA at different concentrations (0, 100, 300µM) in combination with low or high Glc (5 or 25 mmol/L) for 24 hours (Supporting Figure S4A). Afterwards, we tested the effects of the above collected c.m. on untreated HSCs for 24 hours. Interestingly, both concentrations (100 and 300 µmol/L) of PA in combination with 25 mmol/L Glc enhanced significantly LX‐2 migration, whereas 5 mmol/L Glc induced HSCs migration only in combination with the maximum PA dose (300 µmol/L, *P* < .01, Figure 5A). In order to investigate the potential mechanism underlying this effect in LX‐2, we performed a wide array cytokine screening comparing cell culture supernatant collected from HSCs exposed to hepatocytes c.m. (300 μmol/L PA + 25 mmol/L Glc) for 24 hours. Interestingly, exposure to the above IHH c.m. enhanced significantly the release of granulocyte‐macrophage colony‐stimulating factor (GM‐CSF), granulocyte colony‐stimulating factor (G‐CSF) and macrophage migration inhibitory factor (MIF) from HSCs (*P* < .05, Figure 5B) compared to 25 mmol/L Glc alone. In particular, these data align to the increase in PNPLA3 protein level in HSCs, when LX‐2 were exposed to IHH c.m. containing high PA dose (300µM) in combination with Glc (both 5 and 25 mmol/L; *P* < .01, Figure 5C). Notably, activation of JNK pathway was observed when HSCs were exposed to c.m. containing 300µM of PA and 25 mmol/L of Glc (p‐JNK vs total JNK; *P* < .01, Figure 5C), but the increase in autophagy markers LC3A/B and ATG5 was significant only with 5 mmol/L Glc (*P* < .05, Figure 5C).

**Figure 6:**
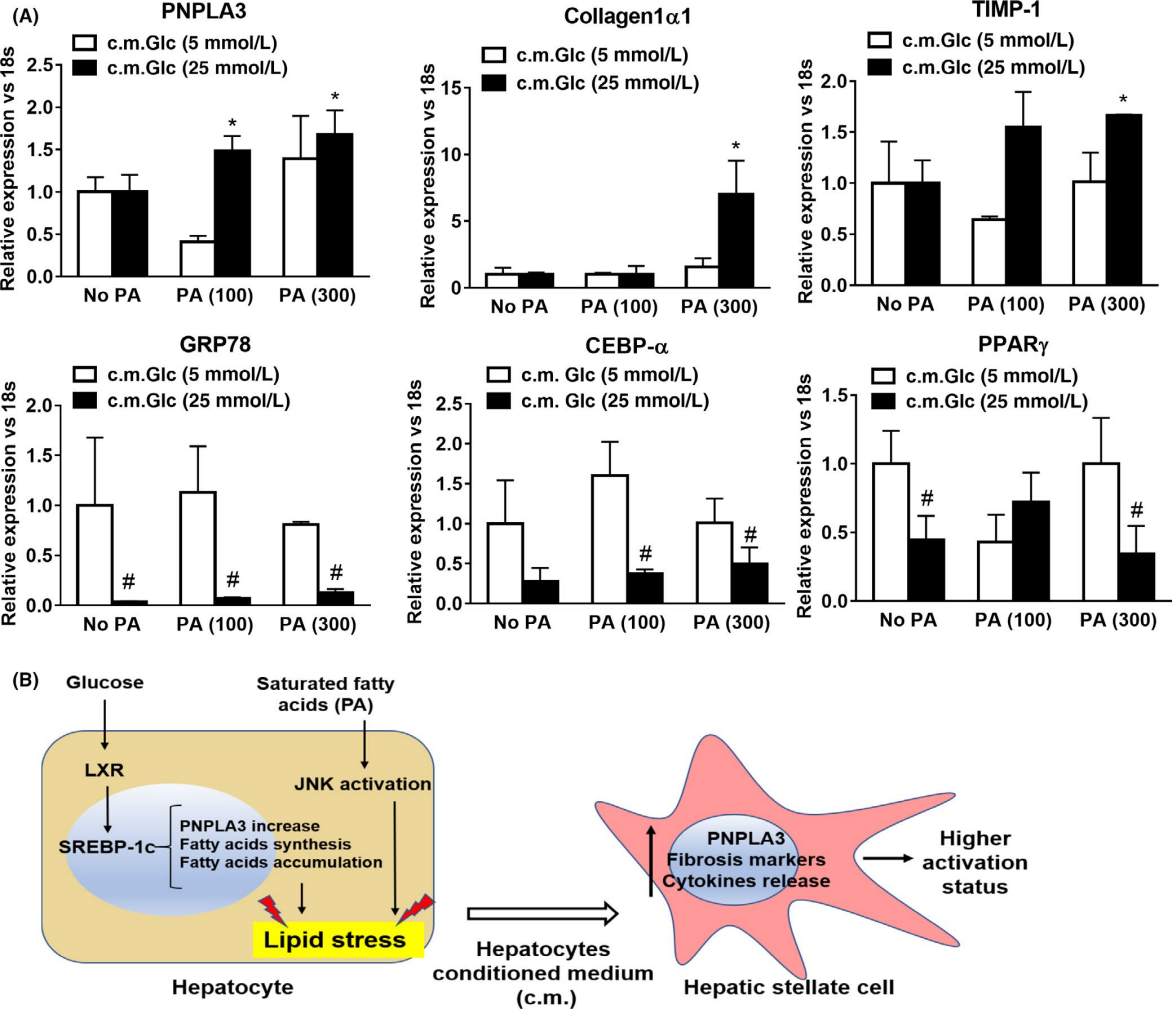
Glucose and Palmitic Acid trigger metabolic stress in hepatocytes, which in turn stimulates increase of PNPLA3, along with autophagic and pro‐fibrotic gene expression and inflammatory cytokine secretion in HSCs. IHH cells cultivated with low (white bars) or high Glc (black bars) alone or in combination with PA (100 and 300 μmol/L). After 24 h, conditioned medium (c.m.) was collected and used to stimulate HSCs for 24 h. Conditions used in IHH cells are described in Material and Methods. A, Expression of PNPLA3, Collagen1α1, TIMP‐1, GRP78, CEBPα and PPARγ analyzed by real‐time PCR and normalized to 18 s. *P < .05 vs c.m. No PA, 25 mmol/L Glc (black bars). #*P* < .01 vs 5 mmol/L Glc (white bars) relative to the same PA concentration. B, Representative scheme resembling PNPLA3 and de‐novo lipogenesis induction through SREBP‐1c and LXR by Glc (25 mmol/L). In addition, saturated FFA such as PA, induce lipid accumulation and cellular stress. Exposure of HSCs to c.m. from lipid stressed hepatocytes results in PNPLA3 increase and aggravates pro‐fibrogenic features (migration, Collagen1α1, α‐SMA, TIMP‐1, cytokine release and autophagic markers) in HSCs

Moreover, gene expression analysis revealed that along with PNPLA3, combination of PA (300 μmol/L) and Glc (25 mmol/L) in IHH c.m. strongly induced in HSCs the expression of two pro‐fibrogenic markers, Collagen1α1 and TIMP‐1 (*P* < .05, Figure 6A), whereas the adipogenic regulators of lipid and glucose metabolism GRP78 and CEBPα were strongly reduced (*P* < .001, Figure 6A), along with the expression of PPARγ and its downstream target CD36 (Figure 6A, Figure S4B).

Collectively, our in vitro model shows that we observed distinct effects in HSCs when exposed to IHH c.m. containing saturated FFA, such as PA, in combination with Glc. In hepatocytes, PNPLA3 expression and protein level are highly induced by Glc via SREBP‐1c and follow the same of the pattern of pro‐lipogenic genes (FASN, ACC‐1, SCD‐1 and LXRα). Glc together with 300 μmol/L of hepatotoxic FFA (PA) induces PNPLA3 expression in hepatocytes thus resulting in increased PNPLA3 transcript and protein level in HSCs, along with higher expression of pro‐fibrotic markers (α‐SMA, Collagen1α1, TIMP‐1), enhanced HSCs migration, activation of JNK pathway and subsequent release of pro‐inflammatory cytokines (Figure 6B).

## 4 DISCUSSION

Our first aim in this study was to evaluate and quantify PNPLA3 expression during different stages of liver fibrosis in NASH patients, according to the different PNPLA3 genotypes (WT = C/C or I148M = G/G). Hereby, we demonstrated that in NASH livers PNPLA3 increased during fibrosis development, from mild (F1) to severe fibrosis (F4), along with the induction of the HSCs activation marker α‐SMA (Figures 1 and 2, Figure S1). Importantly, PNPLA3 also correlates with fibrosis stage and α‐SMA hepatic tissue expression, independently from the genotype (Figure 2B). In addition, PNPLA3 expression is higher in hepatic cells positive for α‐SMA (Figure 2C). Although our patient cohort is relatively small (n = 26) compared to other previous studies, serum liver enzymes such as ALT and AST (Table 1) are consistently increased in NASH patients with the PNPLA3 genetic variant along with the steatosis grade, inflammation, ballooning and NAS score, compared to the C/C carriers (Table 2). Over the last decade the genetic polymorphism of PNPLA3, known as I148M, was strongly linked to non‐alcoholic fatty liver disease (NAFLD)^12^, ^13^, ^14^, ^15^, ^16^, ^37^, ^38^ and to the progression towards severity of liver diseases, such as cirrhosis and liver cancer.^39^, ^40^ Notably, the present study strongly supports our previous findings in vitro with primary human HSCs,^22^, ^23^ and therefore contributes to highlight the importance to group patients according to the different PNPLA3 genotype.

As a second aim, we were interested to explore the potential mechanism underlying the modulation of PNPLA3 expression across different liver cell types, in order to better understand the regulation of this protein under different metabolic stresses (FFAs and Glc) and to better resemble the physiological conditions of patients undergoing liver biopsy. Since patients were starved prior biopsy, we wondered whether the regulation of PNPLA3 expression mediated by glucose already shown in hepatocytes^18^, ^19^ was valid for HSCs as well. Conversely to hepatocytes, here we reported that in HSCs PNPLA3 protein level is high in low glucose concentration (5 mmol/L Glc, Figure 3A,B), whereas induction of PNPLA3 expression is independent from glucose concentration (Figure 3C, 5 mmol/L vs 25 mmol/L = n.s.). This discrepancy between PNPLA3 mRNA and protein levels might be similar to what other groups have been shown previously. Indeed, Huang Y. et al elegantely described that addition of C16:0, C18:0 and C18:1 FFA into culture medium of HuH7 increases and stabilizes PNPLA3 protein levels without altering its transcript.^20^ Moreover, our findings are in line with previous studies, since hepatocytes exposed to 25 mmol/L glucose clearly showed increased amount of the transcription factor SREBP‐1c (Figure 3C) and its transcriptional activity (Figure 3D), which is responsible for PNPLA3 upregulation, along with enhanced expression of SREBP‐1c downstream targets FASN, ACC‐1, SCD‐1 and LXRα. Interestingly, GRP78 reduction observed only in hepatocytes treated with high glucose supports previous findings where reduced expression of GRP78 in the liver was related to hepatic fat accumulation due to decreased inhibition of insulin‐dependent cleavage of SREBP‐1c.^31^, ^32^ In line, hepatocytes CEBPα upregulation with high glucose corresponds to the metabolic induction of hepatic gluconeogenesis.^29^

As third aim, PNPLA3 genetic variant I148M has been strongly associated to progression of liver diseases (NASH) and liver fibrosis development.^12^, ^13^, ^15^, ^16^, ^17^, ^41^, ^42^ In this regard, lipids mediate directly or indirectly activation of intracellular pathways responsible of hepatocellular stress and innate immune system‐mediated inflammation, a condition called lipotoxicity. In particular, the latter term refers to a process by which accumulation of certain toxic lipids, such as saturated FFAs in hepatocytes, triggers various molecular pathways of cell stress (ie JNK pathway), eventually resulting in hepatocellular death and release of molecular mediators affecting neighbouring cells, such as HSCs.^33^ JNK is a member of the mitogen‐activated protein kinases (MAPK) family and it has been shown to play a critical role in dietary animal models of NASH and in human NASH.^43^, ^44^, ^45^ Resembling in vivo data, JNK activation is a recognized hallmark for palmitate‐induced lipotoxicity in hepatocytes in vitro.^36^ To mimic lipid hepatotoxic effects in vitro, we treated hepatocytes with different doses of saturated fatty acid (PA). Lipid accumulation and hepatocellular stress were confirmed by activation of intracellular stress pathways (JNK) and increased expression of PI3KIP1^7^ and IL‐8^35^ (Figure 4A‐C). Importantly, hepatotoxic effects of PA were significantly abolished by the combination with OA (1:2, Supporting Figure S2), in terms of both JNK activation and induction of PI3KIP1 and IL‐8 expression, supporting previous observations on the protective role of OA in hepatocytes.^7^ Interestingly, the exposure of untreated HSCs to lipid stressed hepatocytes c.m. (briefly summarized in Figure 4D) resulted in increased HSCs migration (Figure 4E), significant upregulation of PNPLA3 expression (Figure 4F), in line with the fibrogenic markers Collagen1α1, α‐SMA and TIMP‐1, accompanied by induction of autophagy markers LC3a and ATG5 (Figure 4F). Of note, previous work has reported that TIMP‐1 is highly secreted by LX‐2 overexpressing the I148M variant compared to LX‐2 overexpressing the WT PNPLA3.^46^ This evidence strengthens our findings, since we report that lipid exposed hepatocytes induce PNPLA3 increase and TIMP‐1 expression in HSCs and therefore we might speculate that TIMP‐1 secretion would be more pronounced when HSCs express the I148M PNPLA3.

Conversely, HSCs exposed to c.m. collected from hepatocytes treated with combination of PA and OA (1:2) migrated less and showed reduced PNPLA3 and α‐SMA protein content (Figure S3). The combination of saturated FA (PA) and different doses of glucose determined even more pronounced outcomes in HSCs, as reflected by increased migration, release of cytokines (GM‐CSF, G‐CSF and MIF), PNPLA3 and JNK pathway, along with pro‐fibrogenic genes (Collagen1α1 and TIMP‐1) and downregulation of GRP78, CEBPα and PPARγ (Figures 5 and 6). Interestingly, increased levels of GM‐CSF and G‐CSF were shown to stimulate endogenous mechanisms for hepatic regeneration and tissue repair,^47^ while MIF has been shown to exert antifibrotic effects on HSCs.^48^

In addition to activation of key intracellular pathways, overloaded and damaged hepatocytes release bioactive molecules that interact with target cells and are responsible of the cell‐cell interactions and of the following biological response. Previous studies explored the molecular mechanisms through which lipid overloaded hepatocytes triggered activation of HSCs. Povero and co‐authors demonstrated that lipotoxic‐harmed hepatocytes produce and release extracellular vesicles (EVs) which could carry microRNA molecules able to target PPARγ and therefore promote HSCs activation.^34^ Indeed, our results showed that in HSCs the expression of the PPARγ and its target gene CD36 were reduced under the high glucose and PA levels (Figure 6A, Figure S4B), where pro‐fibrogenic genes Collagen1α1, PNPLA3 and TIMP‐1 were upregulated. Anyhow, further studies will be necessary to explore the mechanism of gene regulation in HSCs via EVs and how glucose and lipotoxic/saturated FFA modulate EVs production in our settings.

Interestingly, the metabolic roles of GRP78 and CEBPα as important regulators of lipid and glucose homeostasis in hepatocytes have been already explored.^29^, ^31^, ^32^ Of note, CEBPα and PPARγ are key transcription factors regulating each other and determining adipose tissue differentiation.^49^ Complex variations in CEBPα, CD36 and PPARγ signalling are thus likely to be explained by the different metabolic requirements of HSCs in response to glucose and FFA release by hepatocytes. PPARγ agonists glitazones are known to reduce lipid stress in the liver,^50^ probably by helping hepatocytes to cope with lipotoxic FFAs stored as lipid droplet thus attenuating the deleterious influence of hepatocytes on HSCs. Moreover, a direct stimulation of PPARγ signalling in HSCs from PNPLA3 I148M patients, where PPARγ signalling is attenuated,^22^ would translate into a synergic therapeutic effect perhaps explaining the beneficial effects obtained on fibrosis in NASH cohorts treated with PPARγ agonist.^50^

In conclusion, these observations emphasize that PNPLA3 expression in human biopsies is closely linked with HSCs activation and liver fibrosis progression in NASH livers, being metabolically controlled by glucose and pro‐steatotic conditions. Moreover, the presence of the genetic variant I148M contributes to characterize the stage and severity of hepatic fibrosis, thus supporting PNPLA3 genotype evaluation as a gold standard for better and specific prognosis. All together, we stress that our preliminary data on in vivo PNPLA3 expression during liver fibrosis progression require future validations also in non‐metabolic hepatic disorders.

## CONFLICT OF INTEREST

Dr Trauner received speaker fees from Bristol‐Myers Squibb (BMS), Falk Foundation, Gilead, Intercept and Merck Sharp & Dohme (MSD); advisory board fees from Albireo, BiomX, Falk Pharma GmbH, GENFIT, Gilead, Intercept, MSD, Novartis, Phenex, and Regulus; travel grants from AbbVie, Falk, Gilead, and Intercept; and unrestricted research grants from Albireo, CymaBay, Falk, Gilead, Intercept, MSD, and Takeda. He is also coinventor of patents on the medical use of norUDCA filed by the Medical University of Graz. All the other authors declare no conflicts of interest.
