# Supplementary figures and images for: Metabolic regulation of hepatic PNPLA3 expression and severity of liver fibrosis in patients with NASH
Source: Liver Int. 2020 Mar 1;40(5):1098–110. doi: 10.1111/liv.14402 (PMC7318357; doi:10.1111/liv.14402)

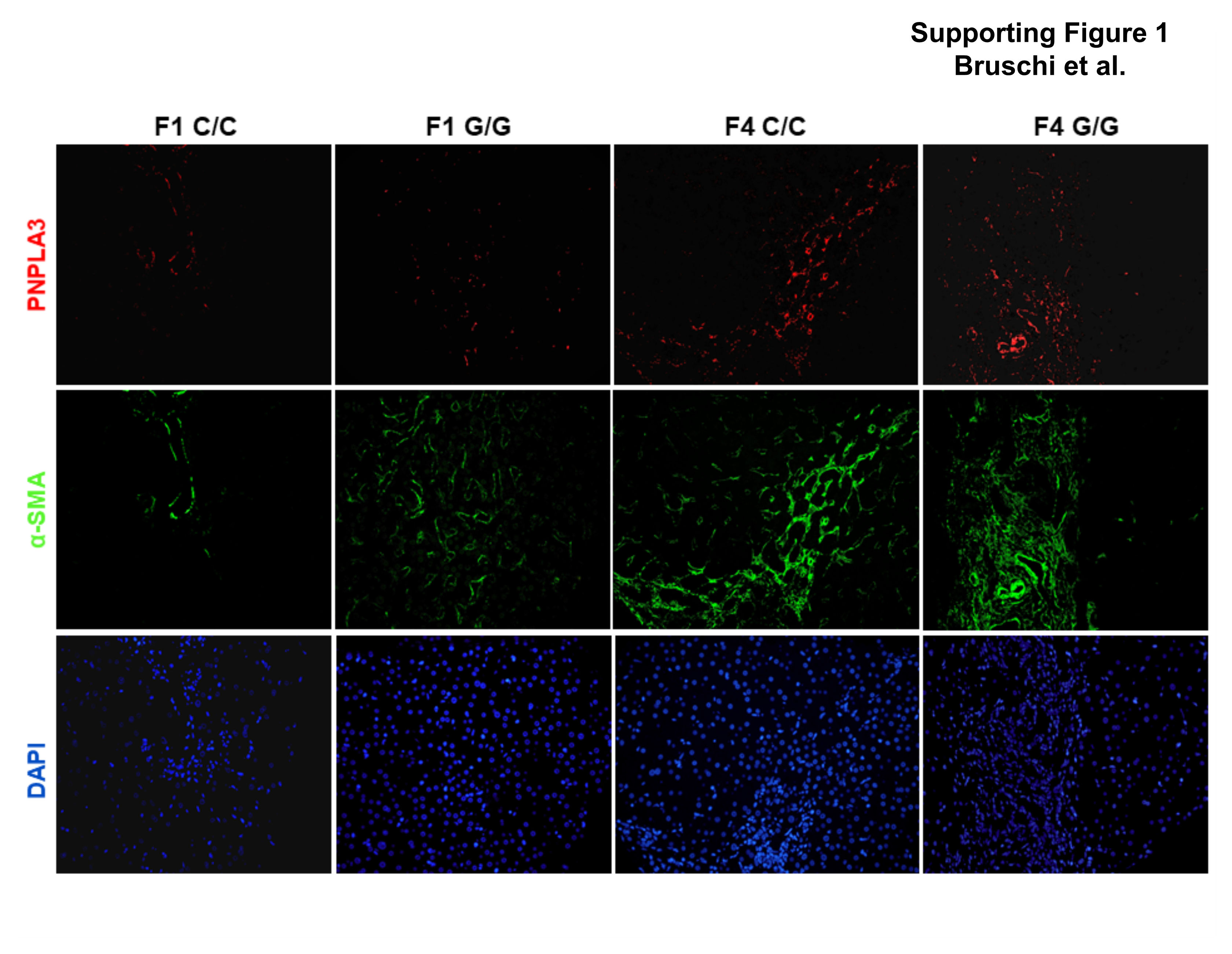

Supplement: Supplementary file 1 [file LIV-40-1098-s001.tif]

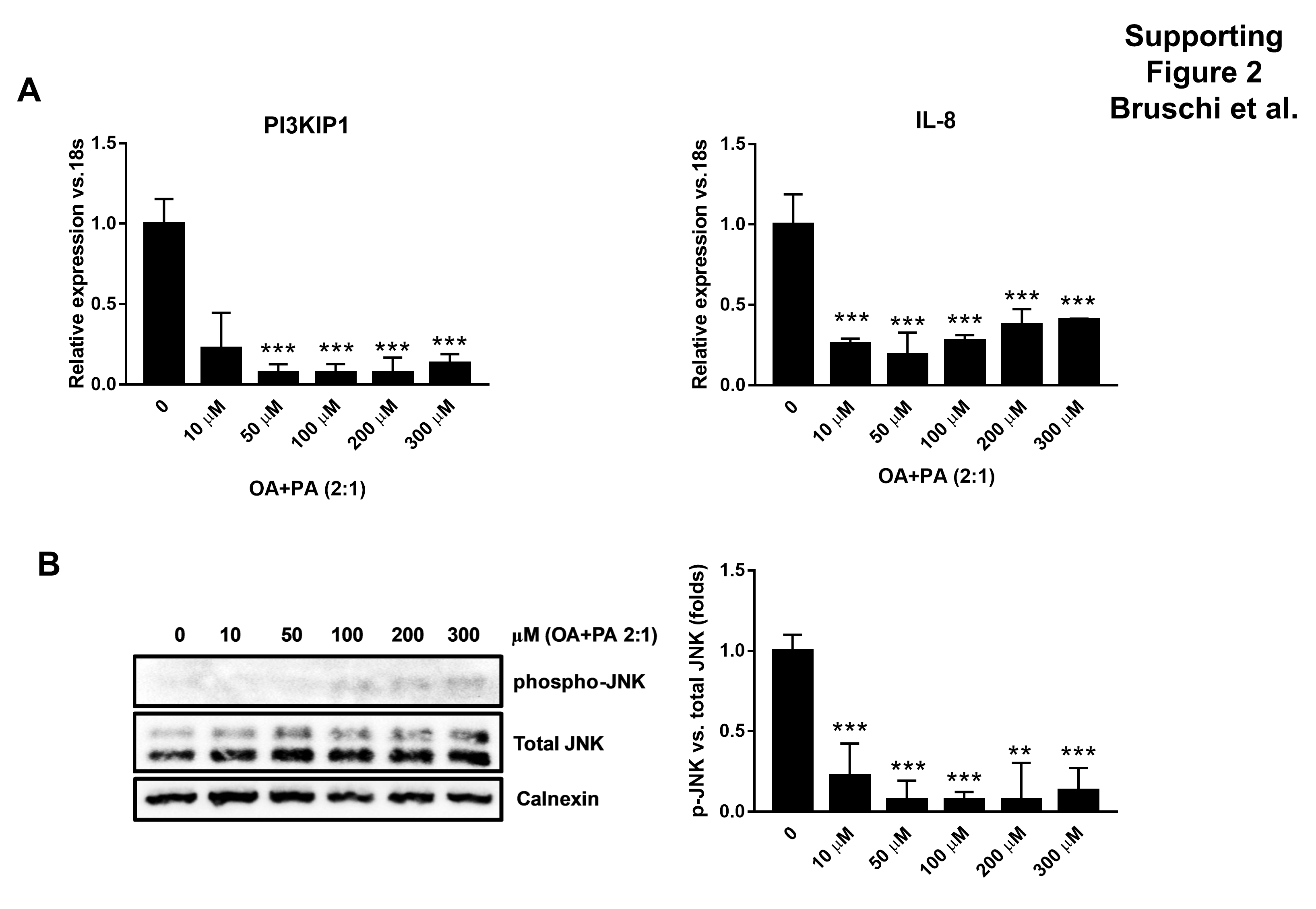

Supplement: Supplementary file 2 [file LIV-40-1098-s002.tif]

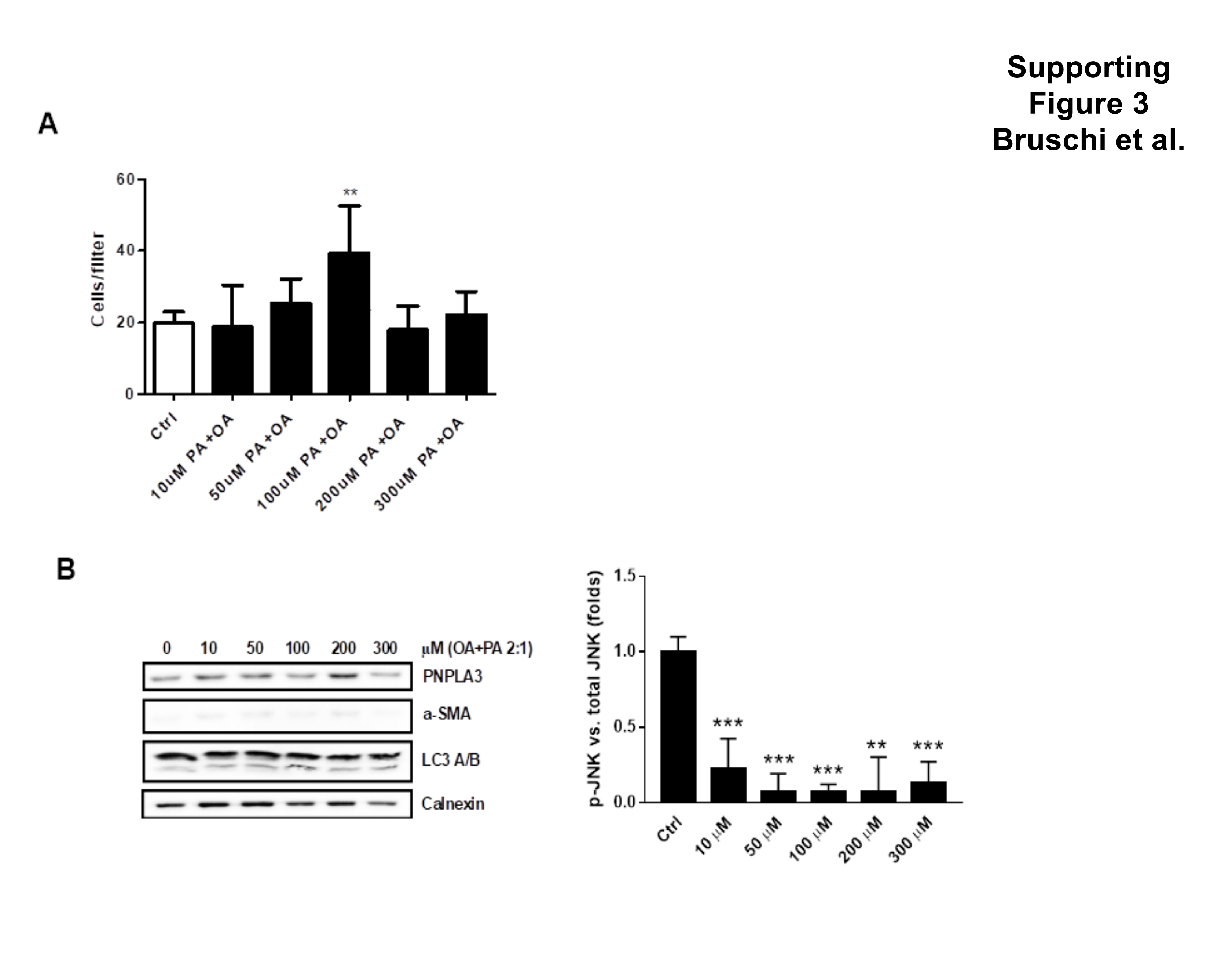

Supplement: Supplementary file 3 [file LIV-40-1098-s003.tif]

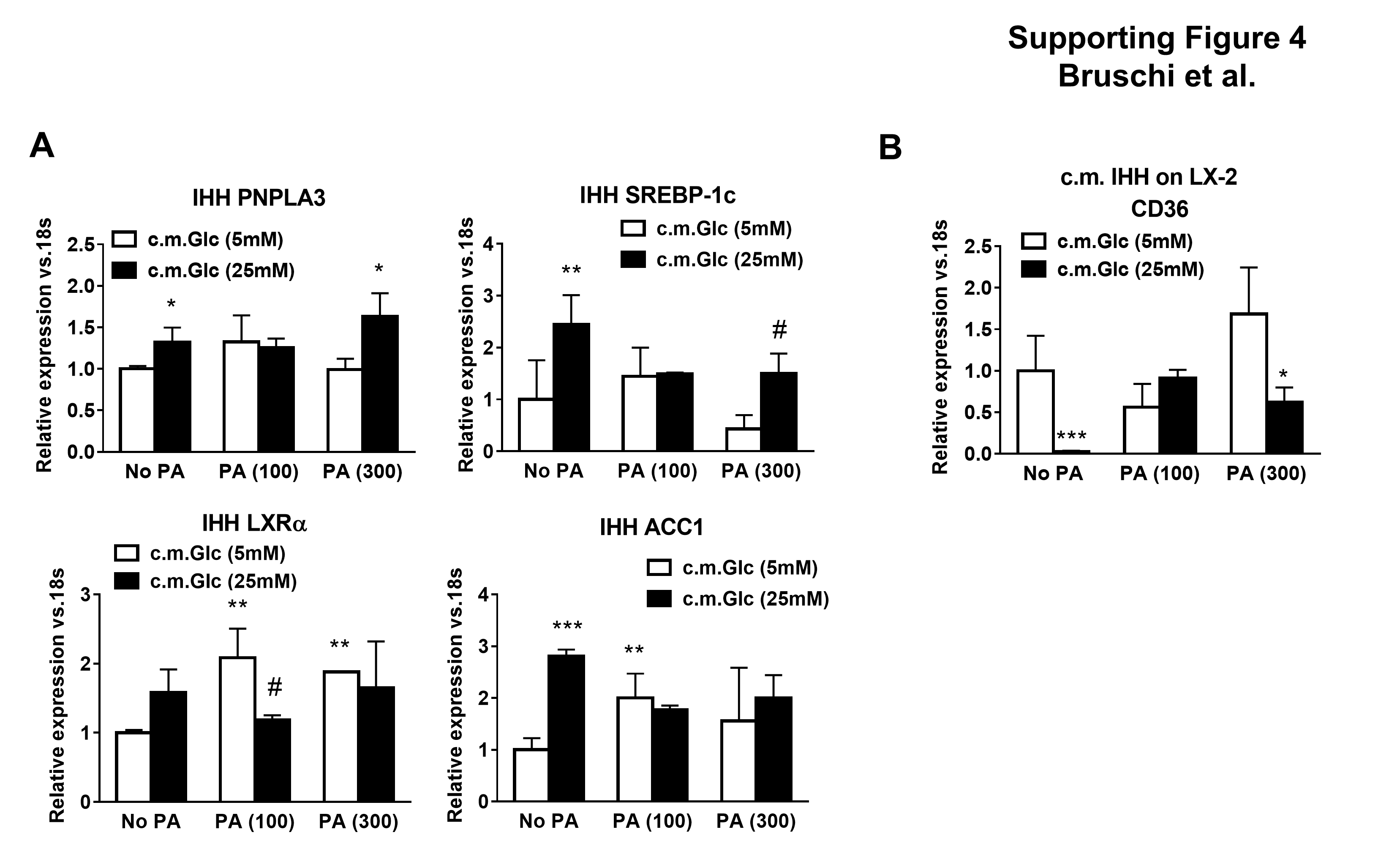

Supplement: Supplementary file 4 [file LIV-40-1098-s004.tif]
